# Fe, N, S-codoped carbon frameworks derived from nanocrystal superlattices towards enhanced oxygen reduction activity

**DOI:** 10.1186/s40580-019-0174-5

**Published:** 2019-02-02

**Authors:** Jinxiang Zou, Biwei Wang, Baixu Zhu, Yuchi Yang, Wenqian Han, Angang Dong

**Affiliations:** 1 iChem, Shanghai Key Laboratory of Molecular Catalysis and Innovative Materials, and Department of Chemistry, Fudan University, Shanghai, 200433 China; 2 State Key Laboratory of Molecular Engineering of Polymers, and Department of Macromolecular Science, Fudan University, Shanghai, 200433 China

**Keywords:** Self-assembly, Nanocrystals, Heteroatom doping, Single atom, Oxygen reduction reaction

## Abstract

Recently, iron, nitrogen and sulfur codoped carbon-based materials have gained increasing attention for their synergistic effect towards superior electrocatalytic oxygen reduction performance. To gain insight into the contributions of the heteroatoms, we developed a facile and reproducible method for constructing Fe, N, S-codoped carbon frameworks derived from self-assembled Fe_3_O_4_ nanocrystal superlattices. The material constructed by the suggested method exhibited excellent ORR activity with more positive half-wave potential (∼ 0.869 V, vs RHE), higher diffusion-limiting current density (∼ 5.88 mA/cm^2^) and smaller Tafel slope (45 mV/dec) compared with Fe, N-codoped carbon frameworks and Pt/C. Notably, Fe_3_O_4_ nanocrystals served as both the building blocks for constructing carbon frameworks and the source of Fe residues leaving in the frameworks at the same time. By artificially tailoring the doping type and level as well as the homogeneousness of heteroatoms, the results discussed herein prove the importance of each kind of heteroatom in boosting ORR activity.

**Electronic supplementary material:**

The online version of this article (10.1186/s40580-019-0174-5) contains supplementary material, which is available to authorized users.

## Introduction

To mitigate environmental problems, more and more efforts have been devoted into searching desired green energy, among which fuel cells and metal-air batteries show tremendous potential [1, 2]. However, the energy efficiency is greatly hindered by the cathodic oxygen reduction reaction (ORR) due to the intrinsic sluggish kinetics [3]. By far, Pt/C and Pt-based noble metal materials are still commonly used as the best commercial ORR catalyst [4]. The drawbacks of Pt-based catalysts, such as the scarcity, high cost, poor durability, and low methanol crossover tolerance, have greatly motivated the research on metal-free and nonprecious-metal-based ORR catalysts in the last few years [5, 6].

Novel carbon-based materials with high surface area, structural stability, as well as morphological diversity have been studied extensively in the field of electrochemical research [7–9]. In particular, the introduction of transition metal atoms (e.g., Fe, Co and Ni) and nonmetal heteroatoms (e.g., N, S, P and B) has been proved to be efficacious in endowing these carbon-based materials with superior ORR catalytic activity, which is attributed to the change in charge and spin densities and increasing defects of the carbon matrix [10–12]. In another word, the electrocatalytic activity for ORR can be artificially tailored by rational design via screening the type of heteroatoms, the doping level, and other relevant factors.

Recently, iron, nitrogen and sulfur codoped carbon-based materials have gained increasing attention for its synergistic effect. For example, sulfur-doped Fe/N/C nanosheets [13], porous Fe–N–S/C catalyst [14] and Fe_1–x_S/Fe_3_O_4_/N, S-doped porous carbon [15] have been designed and studied. The unique nature of S enables the modification of the electronic structure of iron and nitrogen codoped carbon materials, which leads to the boost of the ORR reactivity [16–18]. However, miscellaneous Fe species (e.g., Fe-based sulfides, carbides, and oxides) exist in most of these catalysts complicating the process of verifying heteroatom-induced performance differences. Thus, to gain insight into the roles of heteroatoms, it is of great significance to place more emphasis on designing ORR catalysts with homogeneously dispersed doping atoms.

In this work, we developed a facile and reproducible method for constructing Fe, N, S-codoped carbon frameworks (denoted as Fe–N–S/CFs) via acid etching of Fe_3_O_4_ superlattices derived from self-assembled nanocrystals and subsequent heteroatom-doping with thiourea. Notably, Fe atoms still remained well dispersed rather than agglomeration after doping treatment at the temperature of 900 °C. Compared with Fe, N-codoped carbon frameworks (denoted as Fe–N/CFs) prepared by the same method, Fe–N–S/CFs possessed more competitiveness towards enhanced oxygen reduction reactivity due to the unique structure. When evaluated as electrocatalyst on glassy carbon electrode in alkaline conditions, Fe–N–S/CFs exhibited excellent ORR activity in terms of onset potential, half-wave potential and long-term durability, which outperformed Fe–N/CFs and even commercial Pt/C catalyst (20 wt%). RRDE measurements and Tafel analysis also manifest the facile ORR kinetics of Fe–N–S/CFs. Further results prove the effectiveness of tailoring electrocatalytic activity by controlling the doping level of heteroatoms.

## Results and discussion

### Fabrication procedure

**Scheme 1:**
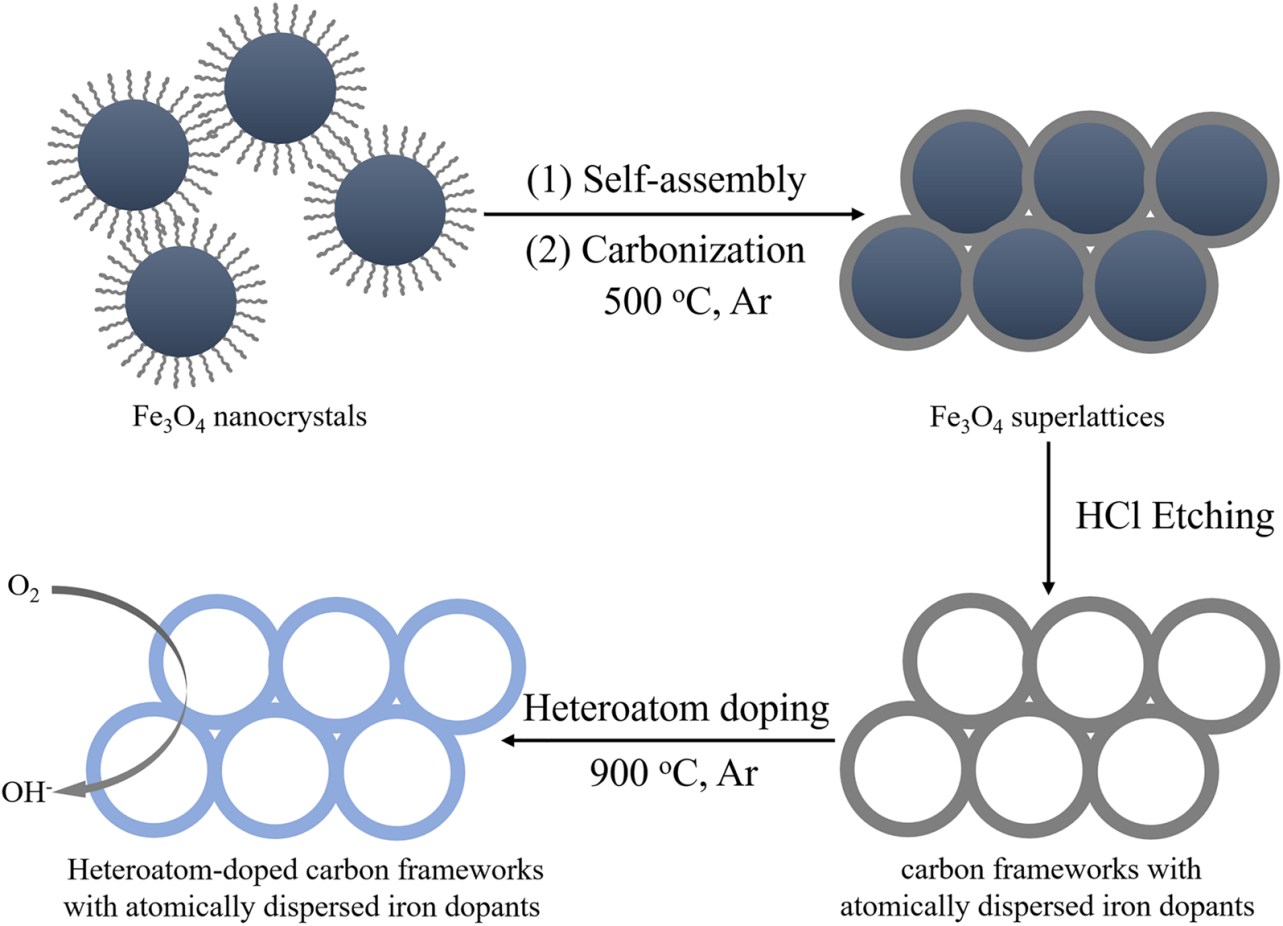
Fabrication procedure of heteroatom-doped carbon frameworks

The fabrication procedure of heteroatom-doped carbon frameworks electrocatalyst is illustrated in Scheme 1. In our previous work, it has been proved that the highly ordered carbon frameworks with atomically dispersed iron dopants can be produced via the transformation of the superlattices of metal oxide nanocrystals, in which the interconnected ultrathin spherical pore walls are derived from the oleic acid (OA) ligands originally stabilizing nanocrystals [19, 20]. Notably, Fe_3_O_4_ nanocrystals served as both the building blocks for constructing carbon frameworks and the source of Fe residues leaving in the frameworks at the same time.

**Fig. 1:**
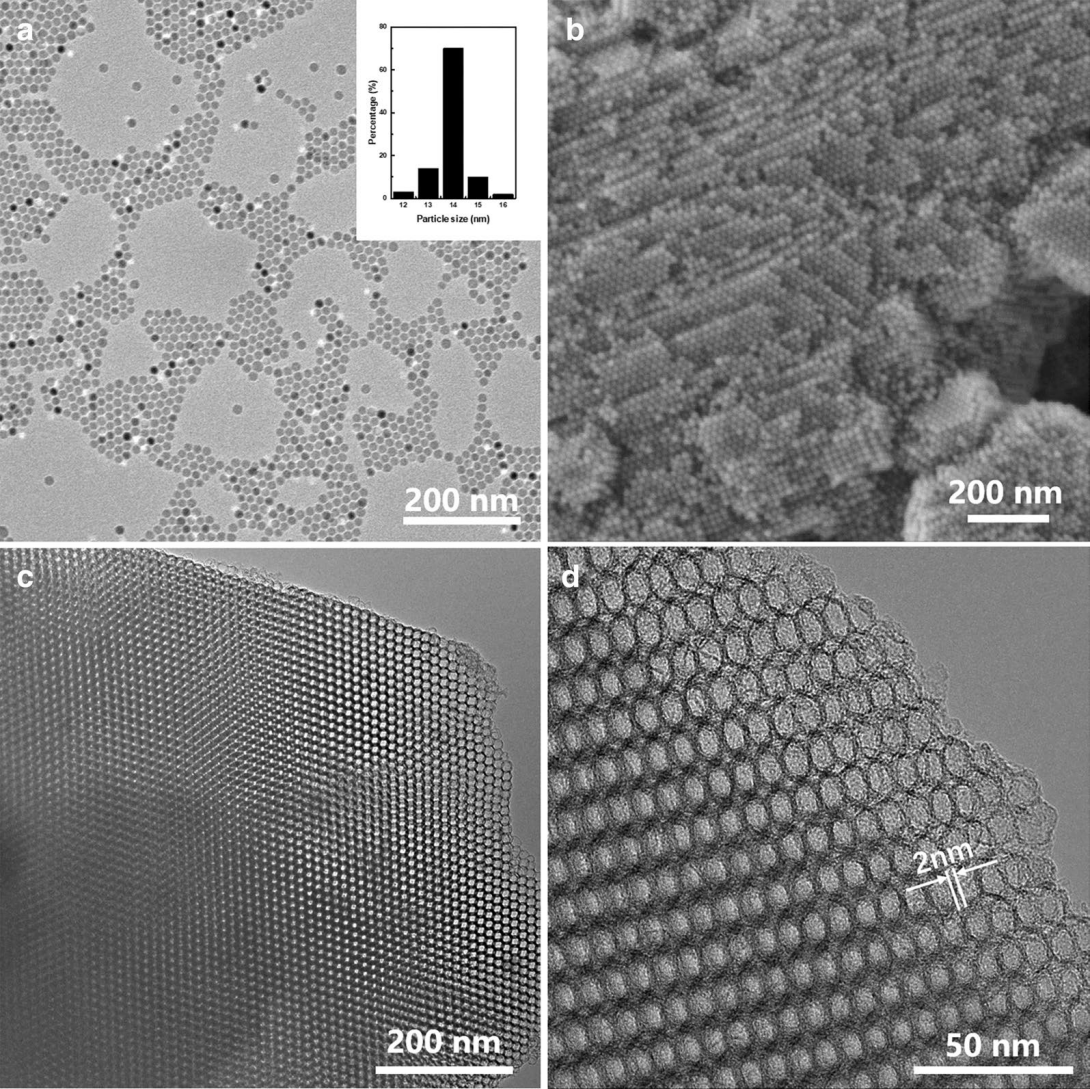
**a** TEM image of monodispersed Fe_3_O_4_ nanocrystals and size distribution analysis (insert). **b** SEM image of ordered Fe_3_O_4_ superlattices via drying-induced self-assembly. **c** TEM image of ordered carbon frameworks after repeated acid treatment. **d** HRTEM image of Fe/CFs which illustrates the ultrathin interconnected spherical pore walls

Figure 1a shows a typical transmission electron microscope (TEM) image of monodispersed Fe_3_O_4_ nanocrystals synthesized by the thermal decomposition of iron-oleate complex, exhibiting an extensive two-dimensional assembly behavior. The size distribution analysis (Fig. 1a insert) was applied to reflect the uniformity of the nanocrystals, which is of great significance to realize ordered assembly. After drying-induced self-assembly, heat treatment at 500 °C in Ar atmosphere for 2 h led to the in situ carbonization of OA ligands covering the surface of nanocrystals, yielding long-range ordered Fe_3_O_4_ superlattices as evidenced by scanning electron microscope (SEM, Fig. 1b). The crystal phase of Fe_3_O_4_ spheres was confirmed by X-ray diffraction (XRD, Additional file 1: Figure S1a), which corresponds to PDF No. 72-2303. Repeated acid treatment with HCl was adopted to remove the building blocks, Fe_3_O_4_ nanocrystals, leaving structurally intact three-dimensional carbon frameworks (denoted as Fe/CFs) instead of the occurrence of collapse. TEM images (Fig. 1c) and small-angle X-ray scattering (SAXS, Additional file 1: Figure S1c) both strongly indicate that the resultant Fe/CFs exhibited a highly ordered mesoporous structure with typical face-centered-cubic (FCC) symmetry. The high-resolution TEM (HRTEM) image reveals that interconnected ultrathin spherical pore walls have a thickness of ~ 2 nm (Fig. 1d).

**Fig. 2:**
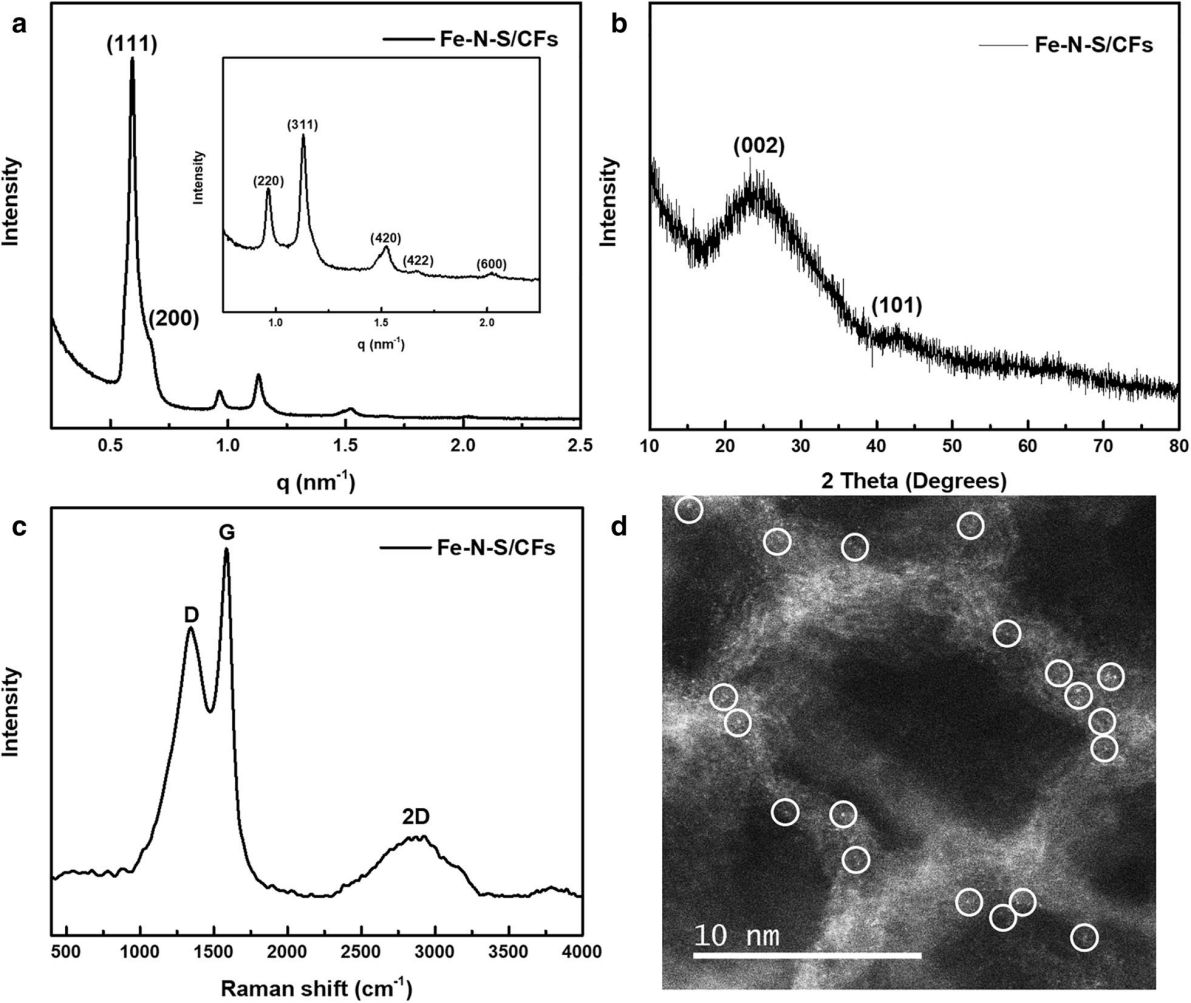
**a** SAXS pattern of highly ordered Fe–N–S/CFs and the enlarged region between 0.75 and 2.25 nm^−1^ (insert). **b** XRD pattern and **c** Raman spectrum of Fe–N–S/CFs. **d** Representative Cs-corrected HAADF-STEM image of Fe–N–S/CFs (the bright spots, assigned to Fe atoms, are circled in the image)

To obtain heteroatom-doped carbon frameworks, Fe/CFs were mixed with doping precursors with the mass ratio of 1:10 and then annealed at 900 °C. Fe–N–S/CFs exhibited almost no apparent morphological changes after doping treatment, remaining highly ordered structure inherited from Fe/CFs evidenced by TEM (Additional file 1: Figure S1d) and SAXS (Fig. 2a). Low-magnification SEM shows that the size of the as-obtained framework particles is in the micrometer scale (Additional file 1: Figure S1e). Two diffraction peaks at about 25° and 43°, assigned to the (002) and (101) plane of graphitic carbon, can be observed in the XRD (Fig. 2b) [21]. The well-resolved G and 2D bands in the Raman spectrum (Fig. 2c) indicate the high graphitization degree of Fe–N–S/CFs [22]. Notably, diffraction peaks corresponding to Fe-based compounds (e.g., metallic iron and iron oxide, carbide, nitride or sulfide) were nearly undetectable. However, there were still trace amounts of Fe signals (~ 0.14 wt%) detectable in energy-dispersive X-ray spectroscopy (EDS, Additional file 1: Figure S1f and Table S1). To ascertain the state of Fe dopants, Fe–N–S/CFs were further characterized by aberration-corrected scanning transmission electron microscope (AC-STEM). Owing to the obvious distinction of the atomic number between Fe and C, the brighter spots, assigned to Fe atoms and well dispersed in the atomic range, were clearly observed in the high-angle annular dark field (HAADF) mode (Fig. 2d). These results suggest that the Fe residues are atomically dispersed on the as-formed carbon frameworks. Fe–N/CFs show similar structural characterizations results as evidenced by Additional file 1: Figure S1 and Table S1.

**Fig. 3:**
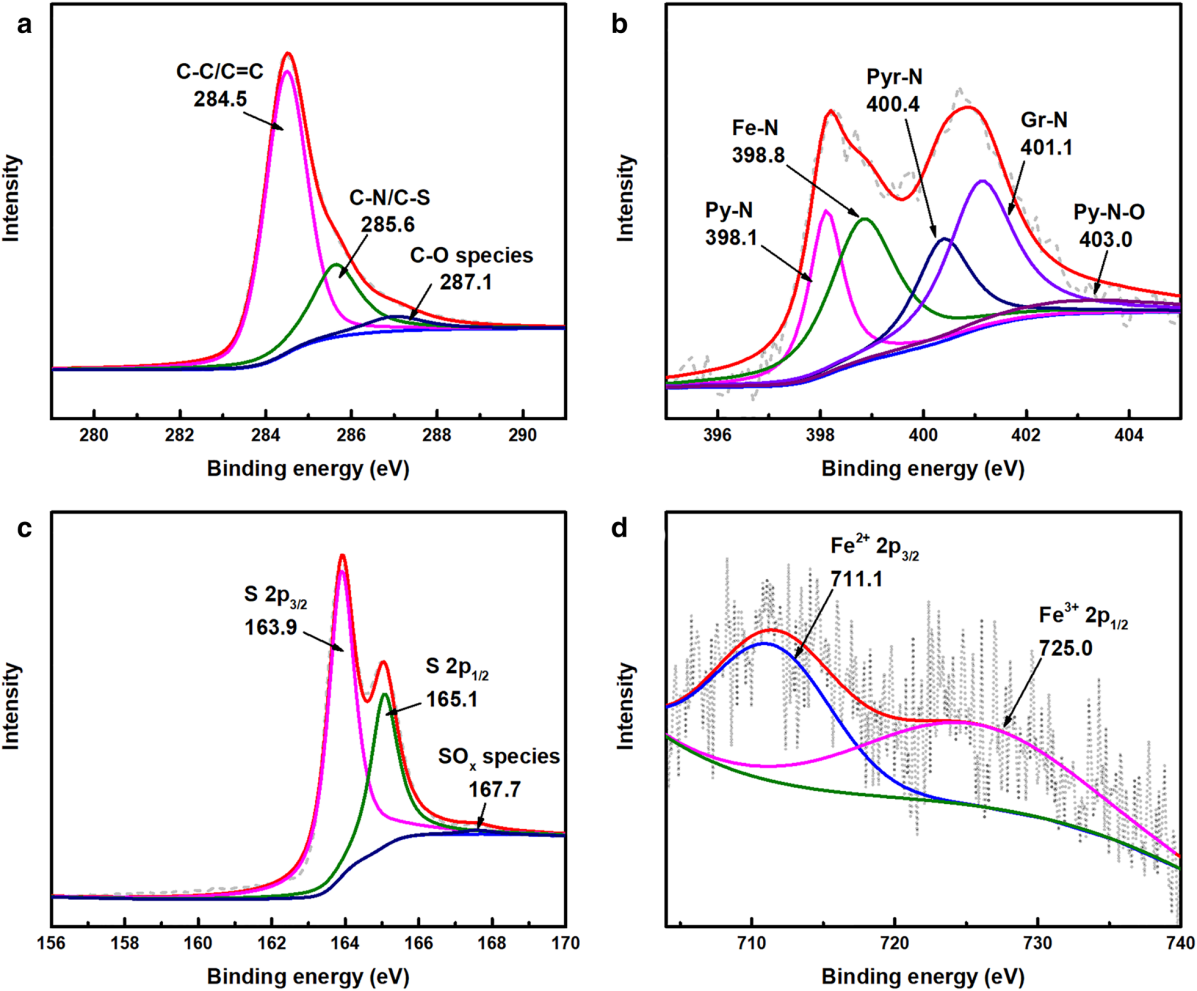
High-resolution **a** C 1s, **b** N 1s, **c** S 2p and **d** Fe 2p XPS spectra of Fe–N–S/CFs

The chemical composition of our materials was analyzed by X-ray photoelectron spectroscopy (XPS). The peak corresponding to 285.6 eV in the fitted C 1s spectrum (Fig. 3a) is good evidence for nitrogen connecting with carbon matrix and C–S bonds also partially contribute to this peak [23]. The spectrum of N 1s (Fig. 3b) can be divided into five characteristic peaks at 398.1, 398.8, 400.4, 401.1, and 403.0 eV, which are assigned to pyridinic nitrogen (Py–N), iron-coordinated nitrogen (Fe–N), pyrrolic nitrogen (Pyr–N), graphite-like nitrogen (Gr–N), and oxidized nitrogen (Py–N–O) nitrogen atoms, respectively [24, 25]. The high-resolution S 2p spectra in Fig. 3c highlights two peaks at binding energies of 163.9 and 165.1 eV, assigned to the S 2p_3/2_ and S 2p_1/2_ states of sulfide species (C–S–C) [13]. Notably, doping of sulfur atoms leads to thiophene-like structures with neighboring carbon atoms, which may cause positively charged carbon atoms to favor adsorption of oxygen species [17, 26]. Meanwhile, a weak SO_x_ species peak with higher binding energy can also be observed [27]. Two peaks centered at 711.1 and 725.0 eV are observed in Fig. 3d, which match well with Fe^2+^ 2p_3/2_ and Fe^3+^ 2p_1/2_, respectively, consistent with previous results [28]. The detailed XPS results of nitrogen-doped carbon frameworks are shown in Additional file 1: Figure S2. It is believed that Fe–N and Gr–N are the main active sites for ORR, ratios of which in Fe–N–S/CFs are determined to be 1.07 at% and 1.08 at%, respectively, higher than that in Fe–N/CFs (determined to be 0.46 at% and 0.77 at%, respectively, Additional file 1: Table S2) [29, 30]. The EDS-mapping results of Fe–N–S/CFs (Additional file 1: Figure S1f) further prove the uniform dispersion of Fe, N and S in carbon frameworks. Notably, there is a trace amount of Cl detected in Fe–N/CFs by EDS (Additional file 1: Figure S2d). According to previous results, the introduction of Cl originated from the decomposition of NH_4_Cl shows no negative effects on catalytic activity [31]. Based on the above results, it is reasonable to speculate Fe–N–S/CFs can be a competitive candidate towards electrocatalytic oxygen reduction.

### Electrocatalytic performance

The successful fabrication of different heteroatom doping electrocatalysts with unique structure, including highly ordered mesoporosity, atomically dispersed Fe dopants and well-retained carbon frameworks, allows a detailed study on the role of heteroatoms and their intrinsic ORR activities.

**Fig. 4:**
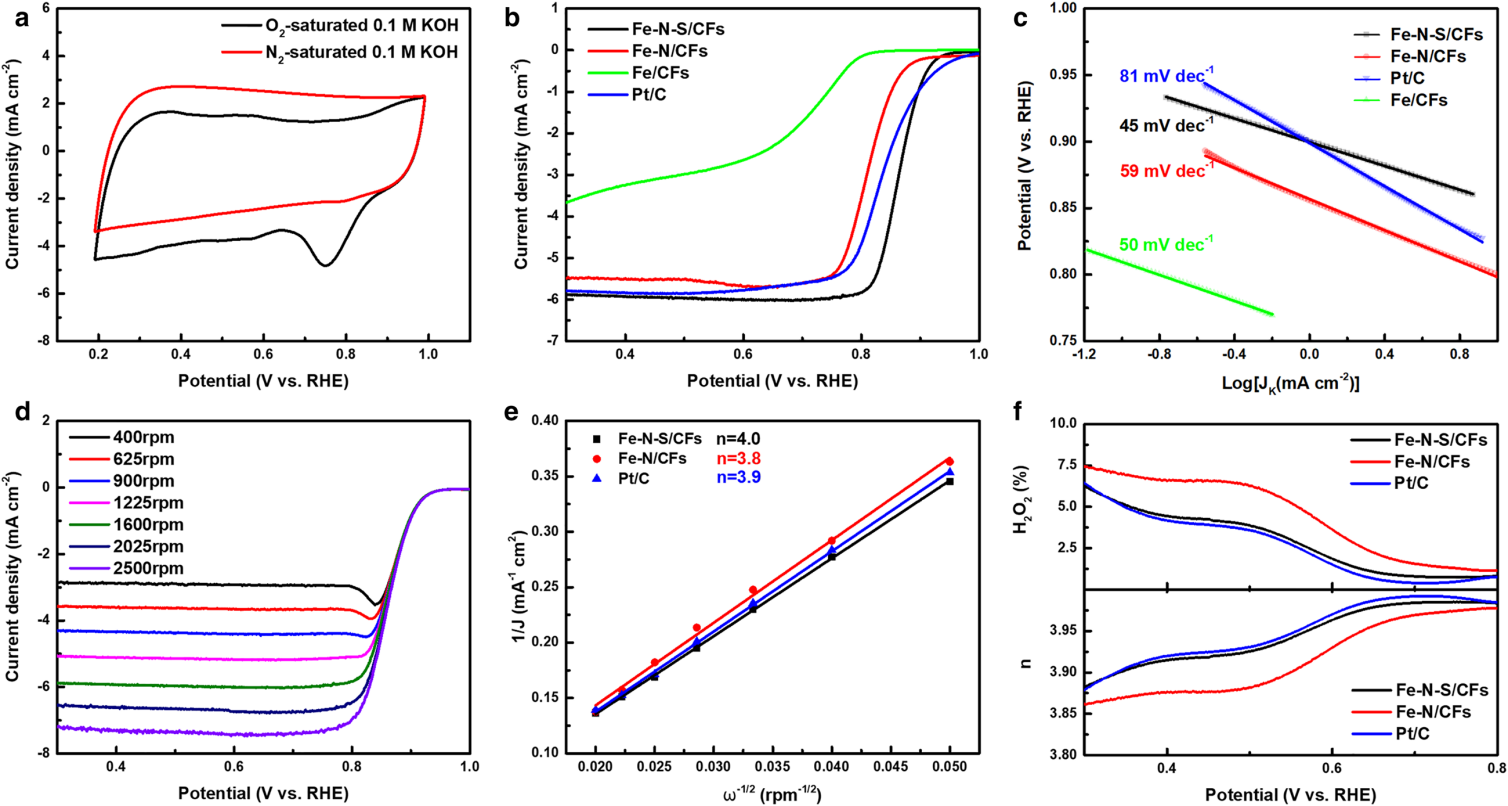
**a** CV curves of Fe–N–S/CFs tested in O_2_ or N_2_ saturated 0.1 M KOH. **b** LSV curves of Fe–N–S/CFs, Fe–N/CFs, Fe/CFs and Pt/C tested at the rotating speed of 1600 rpm. **c** Tafel plots of Fe–N–S/CFs, Fe–N/CFs and Pt/C measured at the rotating speed of 1600 rpm. **d** LSV curves of Fe–N–S/CFs with rotating speed ranging from 400 to 2500 rpm. **e** K–L plots of Fe–N–S/CFs, Fe–N/CFs and Pt/C at 0.4 V vs. RHE. **f** H_2_O_2_ yielding and electron transfer number curves of Fe–N–S/CFs, Fe–N/CFs and Pt/C

The ORR catalytic performance of the as-prepared electrocatalysts was first evaluated by rotating disk electrode (RDE) measurement. Cyclic voltammetry (CV) curve of Fe–N–S/CFs (Fig. 4a) shows a distinct peak centered at 0.75 V (vs. RHE) in O_2_-saturated 0.1 M KOH, whereas a featureless curve is observed in N_2_-saturated solution, preliminarily suggesting its electrocatalytic efficiency. Compared with Additional file 1: Figure S3a, the results further indicate that Fe–N–S/CFs exhibit better ORR performance, peak center of which is at least 80 mV more positive than that of Fe–N/CFs. The linear sweep voltammetry (LSV) results (Fig. 4b) also manifest the superior electrocatalytic activity of Fe–N–S/CFs, which exceeds Fe–N/CFs and even commercial 20 wt% Pt/C catalyst, as significantly indicated by the more positive half-wave potential (∼ 0.869 V, vs RHE) and the higher diffusion-limiting current density (5.88 mA/cm^2^). Moreover, Fe–N–S/CFs also possessed the smallest Tafel slope (45 mV/dec, Fig. 4c), revealing its facile ORR kinetics. Notably, without nonmetal heteroatoms doping, Fe/CFs exhibited very poor ORR catalytic activity (Fig. 4b, green curve), which clearly proves the indispensability of introducing N and S in obtaining high ORR activity.

To gain further insight into the catalytic process, LSV measurements at different electrode rotation speed were recorded. As shown in Fig. 4d, the diffusion-limiting current density of Fe–N–S/CFs increased with the speed increasing, which is attributable to the improved oxygen diffusion behavior [32]. Both of the heteroatom-doped carbon frameworks show good linearity in Koutecky–Levich (K–L) plots (Fig. 4e), suggesting the first-order oxygen reduction kinetics [33]. Based on the K–L equation, electron transfer number (n) of Fe–N–S/CFs is calculated to be 4.0, close to the theoretical value of ideal catalyst following 4e reduction pathway and higher than the value of Fe–N/CFs (calculated to be 3.8). Rotating ring-disk electrodes (RRDE) measurement was adopted to further quantify the materials’ ORR efficiency. Remarkably, although Fe–N–S/CFs exhibited larger reaction current on the disk, the peroxide yield is still over 1% less than sulfur-free Fe–N/CFs at 0.3 V vs. RHE (Fig. 4f). It clearly indicates that a higher proportion of oxygen is directly reduced into OH^−^ without intermediate peroxides on Fe–N–S/CFs, which corresponds with values of n calculated by RRDE results (Fig. 4f). The above comparison of the ORR performance can strongly prove the introduction of S dopants synergistic with Fe–N–C is an ideal method to enhance oxygen reduction reactivity.

Apart from the high activity and efficiency for ORR, Fe–N–S/CFs also show excellent long-term stability as indicated by chronoamperometric measurements. As shown in Additional file 1: Figure S3d, Fe–N–S/CFs can maintain a high current retention of 98% after 48,000 s of continuous operation (0.7 V vs. RHE) in O_2_-saturated 0.1 M KOH, higher than that of Fe–N/CFs (93%) and Pt/C (88%) tested under the same conditions. The ordered structure retained in Fe–N–S/CFs after durability test showing the stability of carbon frameworks, as confirmed by TEM image (Additional file 1: Figure S4).

**Fig. 5:**
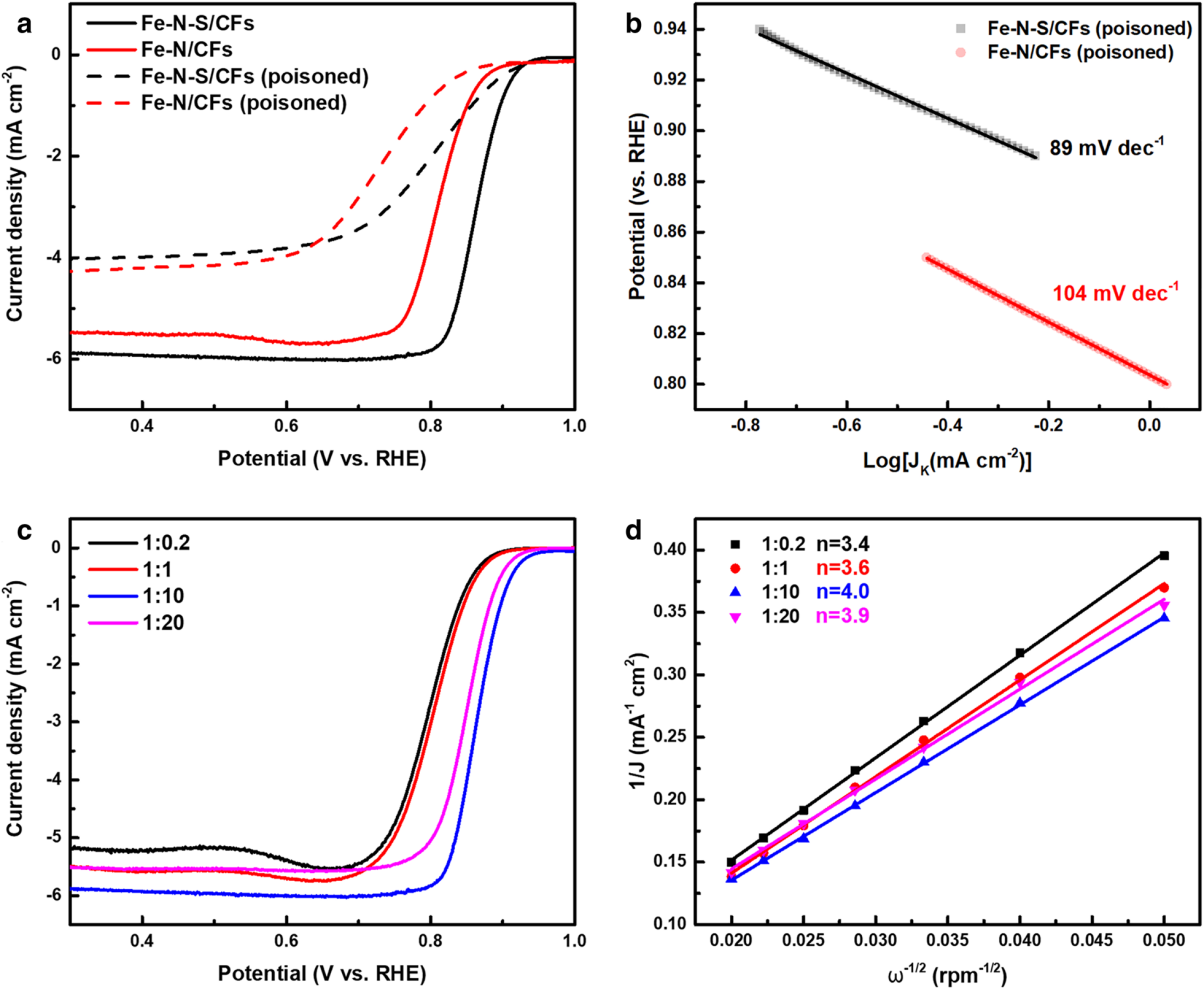
**a** LSV curves of Fe–N–S/CFs and Fe–N/CFs before and after poisoned by SCN^−^ tested at the rotating speed of 1600 rpm. **b** Tafel plots of Fe–N–S/CFs and Fe–N/CFs after poisoned by SCN^−^. **c** LSV curves of N and S codoped carbon frameworks with different doping ratio measured at the rotating speed of 1600 rpm. **d** K–L plots of N and S codoped carbon frameworks with different doping ratio at 0.4 V vs. RHE

To determine the contribution of Fe dopants, Fe–N–S/CFs and Fe–N/CFs were assessed in O_2_-saturated 0.1 M KOH + 5 mM NaSCN solutions. As shown in Fig. 5a, both of the two catalysts exhibited over 60 mV negative shift in half-wave potential and obvious decrease in diffusion-limiting current density. The significant increasing Tafel slopes (Fig. 5b) also indicate the relatively sluggish ORR kinetics. However, the control experiment using commercial 20 wt% Pt/C catalyst shows an unobvious change in ORR activity (Additional file 1: Figure S5). The above results suggest the formation of strong coordinating bonds between SCN^−^ and Fe residues and also demonstrate the pivotal role of Fe dopants in achieving superior catalytic performance.

The doping level of heteroatoms is another factor affecting electrocatalytic performance. Thus, the samples prepared with different mass ratios of carbon frameworks and doping precursors were assessed by RDE measurements (Additional file 1: Figure S6) and K–L analysis. The N and S contents were measured to be 2.96, 3.77, 4.18 and 4.96 wt% and 1.05, 1.65, 2.97 and 4.76 wt%, respectively, when the precursor-to-carbon framework mass ratio is 1:0.2, 1:1, 1:10 and 1:20 (Additional file 1: Table S3). The increasing doping level led to an obvious ORR performance enhancement with the mass ratio ranging from 1:0.2 to 1:10 at the rotating speed of 1600 rpm (Fig. 5c). When the ratio further increases to 1:20, LSV results show almost the same half-wave potential with Fe–N–S/CFs doping with 1:10 ratio. Based on the K–L equation, the values of n are calculated to be 3.4, 3.6, 4.0 and 3.9, respectively with the ratio increasing (Fig. 5d). The enhancement of oxygen reduction reactivity can be ascribed to the increasing content of N and S as evidenced by EDS results. When the amount of thiourea is over ten times higher than carbon frameworks, the increment of heteroatoms would be less efficacious in further lifting oxygen reduction performance.

## Conclusion

In summary, we have demonstrated a facile and reproducible strategy to fabricate Fe, N, S-codoped carbon frameworks derived from self-assembled Fe_3_O_4_ nanocrystal superlattices with superior ORR performance, which outperformed Fe–N/CFs and even commercial Pt/C catalyst. Considering the homogeneous dispersion of heteroatoms, facile tunability of doping type and level as well as detailed study on structure and electrocatalytic reactivity, the results discussed herein provide an important perspective to understand the role of each kind of heteroatom in boosting ORR activity. With further screening relevant factors, the study witnessed a good opportunity to figure out the intrinsic mechanism which is of significance to rationally design a desirable catalyst in the future.

## Experimental

### Chemicals

Oleic acid (OA, 90%),1-octadecene (ODE, 90%) sodium oleate (CP), thiourea (99%) and Nafion (5 wt%, containing 15 ~ 20 wt% water) were purchased from Aldrich. Iron(III) chloride hexahydrate (FeCl_3_·6H_2_O, 99.0%) was purchased from J&K Chemical Co., Ltd. Anhydrous ethanol, isopropanol, ammonium chloride (NH_4_Cl), sodium sulfocyanate (NaSCN) and hexane were obtained from Sinopharm Chemical Reagent Co., Ltd (China). All chemicals were used as received without further purification.

### Synthesis of Fe_3_O_4_ nanocrystals

Monodispersed Fe_3_O_4_ nanocrystals were synthesized according to a literature method [34]. Firstly, iron oleate was obtained by the reaction between FeCl_3_·6H_2_O and sodium oleate. In a typical synthesis for 14 nm Fe_3_O_4_ nanocrystals, 18 g of iron oleate and 4.3 g of OA were dissolved in 120 g of ODE in a three-neck flask. The mixture was degassed under vacuum at 120 °C for 0.5 h, heated up to 320 °C under N_2_ atmosphere and kept at this temperature for 1 h. The as-synthesized Fe_3_O_4_ nanocrystals were precipitated from the reaction solution by addition of isopropanol and ethanol. After centrifugation. The precipitated Fe_3_O_4_ nanocrystals were re-dispersed in hexane with a suitable concentration.

### Fabrication of heteroatom-doped carbon frameworks

Fe_3_O_4_ superlattices were obtained by the evaporation of the solution containing Fe_3_O_4_ nanocrystals under room condition via drying-induced self-assembly. After following heat treatment at 500 °C in Ar atmosphere for 2 h, repeated acid treatment with HCl was adopted to remove Fe_3_O_4_ nanocrystals. To realize N and S doping, the as-obtained carbon frameworks and thiourea with a mass ratio of 1:10 were mixed and heated up to 900 °C in Ar atmosphere and kept for 1 h. Fe–N/CFs were fabricated in the same way using NH_4_Cl as doping precursor. Doping level was adjusted by changing the mass ratio from 1:0.2 to 1:20.

### Characterization

Scanning electron microscopy (SEM) was taken on a Zeiss Ultra 55 microscope operated at 5 kV. Transmission electron microscopy (TEM), high-resolution TEM (HRTEM), scanning TEM (STEM), energy-dispersive X-ray spectroscopy (EDS) and elemental mapping were conducted by a FEI Tecnai G^2^ F20 S-TWIN microscope operated at 200 kV. Cs-corrected HAADF-STEM measurement was carried out on a Titan G2 60–300 microscope operated at 300 kV. X-ray photoelectron spectroscopy (XPS) was performed on a Perkin Elmer PHI-5000C ESCA system. Small-angle X-ray scattering (SAXS) was conducted on a Nanostar U small angle X-ray scattering system using Cu Ka radiation (40 kV, 35 mA). Raman spectra were recorded at room temperature with an XploRA Raman system.

### Electrochemical tests

All of the catalysts inks were prepared by homogeneously mixing 1 mg of catalyst, 0.25 mg of Carbon ECP, 6 μL of Nafion, and 250 μL of anhydrous ethanol. A certain volume of the ink was dropped on glassy carbon electrode and dried at room temperature. Electrochemical measurements were carried out on CHI 760E electrochemical station in 0.1 M KOH electrolyte. A carbon rod and a saturated calomel electrode (SCE) electrode were selected to be the counter electrode and reference electrode. Oxygen or nitrogen flow was used for certain measurements. Cyclic voltammetry (CV) experiments were recorded at a sweep rate of 50 mV/s, and the Linear sweep voltammetry (LSV) tests were measured with a scan rate of 20 mV/s under various rotation rates.

For the Tafel plot, the kinetic current was calculated from the mass-transport correction of RDE by:

$$J_{K} = \frac{{J \times J_{L} }}{{J_{L} - J}}$$

For RDE measurements, the electron transfer number (n) was determined by the Koutecky–Levich equation:

$$\frac{1}{J} = \frac{1}{{J_{L} }} + \frac{1}{{J_{K} }} = \frac{1}{{B\omega^{1/2} }} + \frac{1}{{J_{K} }}$$

$$B = 0.62nFC_{{O_{2} }} \left( {D_{{O_{2} }} } \right)^{2/3} v^{ - 1/6}$$

where J represents the measured current density, J_K_ and J_L_ are the kinetic and limiting current densities, ω is the angular velocity of the disk, n is the electron transfer number, F is the Faraday constant, ${\text{C}}_{{{\text{O}}_{2} }}$ is the bulk concentration of O_2_ (1.2 × 10^−6^ mol/cm), ${\text{D}}_{{{\text{O}}_{2} }}$ is the diffusion coefficient of O_2_ (1.9 × 10^−5^ cm^2^/s) and v is the kinematic viscosity of the electrolyte (0.01 cm^2^/s).

For RRDE measurements, the hydrogen peroxide yield (H_2_O_2_%) and the electron transfer number (n) were calculated by:

$$\text{H}_{2} \text{O}_{2}\ (\%) = 200 \times \frac{{I_{r} /N}}{{I_{d} + I_{r} /N}}$$

$${\text{n}} = 4 \times \frac{{I_{d} }}{{I_{d} + I_{r} /N}}$$

where I_d_ is the disk current, I_r_ is the ring current and N = 0.37 is current collection efficiency of the Pt ring. The ring electrode potential was set to 1.23 V vs. RHE.

The durability of the catalysts was tested in the O_2_-saturated 0.1 M KOH electrolyte at room temperature by applying chronoamperometric measurements at 0.7 V vs. RHE for 48000 s.

## Additional file

**Additional file 1.** Additional figures and tables.

## References

[1] Wu G, More KL, Johnston CM, Zelenay P (2011). Science.

[2] Cheng F, Chen J (2012). Chem. Soc. Rev..

[3] Debe MK (2012). Nature.

[4] Wang YJ, Zhao N, Fang B, Li H, Bi XT, Wang H (2015). Chem. Rev..

[5] Dai L, Xue Y, Qu L, Choi HJ, Baek JB (2015). Chem. Rev..

[6] Xia ZH, An L, Chen PK, Xia DG (2016). Adv Energy Mater..

[7] Gu WT, Yushin G (2014). WIRES Energy Environ..

[8] Wang Y, Li J, Wei ZD (2018). Chemelectrochem..

[9] Zhang C, Lv W, Tao Y, Yang QH (2015). Energ. Environ. Sci..

[10] Bezerra CWB, Zhang L, Lee KC, Liu HS, Marques ALB, Marques EP, Wang HJ, Zhang JJ (2008). Electrochim. Acta.

[11] Choi CH, Park SH, Woo SI (2012). J. Mater. Chem..

[12] Zhang JT, Dai LM (2015). ACS Catal..

[13] Hu K, Tao L, Liu D, Huo J, Wang S (2016). ACS Appl. Mater. Inter..

[14] Liu X, Chen C, Cheng QQ, Zou LL, Zou ZQ, Yang H (2018). Catalysts.

[15] Zhang JW, Xu D, Wang CC, Guo JN, Yan F (2018). Adv. Mater. Interfaces..

[16] Li Q, Chen W, Xiao H, Gong Y, Li Z, Zheng L, Zheng X, Yan W, Cheong WC, Shen R, Fu N, Gu L, Zhuang Z, Chen C, Wang D, Peng Q, Li J, Li Y (2018). Adv. Mater..

[17] Shen H, Gracia-Espino E, Ma J, Zang K, Luo J, Wang L, Gao S, Mamat X, Hu G, Wagberg T, Guo S (2017). Angew. Chem. Int. Edit..

[18] Wu K, Chen X, Liu S, Pan Y, Cheong W-C, Zhu W, Cao X, Shen R, Chen W, Luo J, Yan W, Zheng L, Chen Z, Wang D, Peng Q, Chen C, Li Y (2018). Nano Res..

[19] Jiao Y, Han D, Ding Y, Zhang X, Guo G, Hu J, Yang D, Dong A (2015). Nat. Commun..

[20] Han D, Jiao Y, Han W, Wu G, Li T, Yang D, Dong A (2018). Carbon.

[21] Jiao Y, Han D, Liu L, Ji L, Guo G, Hu J, Yang D, Dong A (2015). Angew. Chem.-Int. Edit..

[22] Cui C, Qian W, Yu Y, Kong C, Yu B, Xiang L, Wei F (2014). J. Am. Chem. Soc..

[23] Wang Z, Dong Y, Li H, Zhao Z, Bin Wu H, Hao C, Liu S, Qiu J, Lou XW (2014). Nat. Commun..

[24] Serov A, Artyushkova K (2014). P. Atanassov..

[25] Jiang WJ, Gu L, Li L, Zhang Y, Zhang X, Zhang LJ, Wang JQ, Hu JS, Wei Z, Wan LJ (2016). J. Am. Chem. Soc..

[26] Chen P, Zhou T, Xing L, Xu K, Tong Y, Xie H, Zhang L, Yan W, Chu W, Wu C (2017). Angew. Chem.-Int. Edit..

[27] Naveen MH, Shim K, Hossain MSA, Kim JH, Shim YB (2017). Adv. Energy Mater..

[28] Wang B, Wang X, Zou J, Yan Y, Xie S, Hu G, Li Y, Dong A (2017). Nano Lett..

[29] Liu Y, Li J, Li W, Li Y, Chen Q, Liu Y (2015). Int. J. Hydrogen Energ..

[30] Wang H, Maiyalagan T, Wang X (2012). ACS Catal..

[31] Varnell JA, Tse ECM, Schulz CE, Fister TT, Haasch RT, Timoshenko J, Frenkel AI, Gewirth AA (2016). Nat. Commun..

[32] Li T, Xue B, Wang B, Guo G, Han D, Yan Y, Dong A (2017). J. Am. Chem. Soc..

[33] Mosa IM, Biswas S, El-Sawy AM, Botu V, Guild C, Song W, Ramprasad R, Rusling JF, Suib SL (2016). J. Mater. Chem. A..

[34] Park J, An K, Hwang Y, Park JG, Noh HJ, Kim JY, Park JH, Hwang NM, Hyeon T (2004). Nat. Mater..

