# Supplementary material for: Fe, N, S-codoped carbon frameworks derived from nanocrystal superlattices towards enhanced oxygen reduction activity
Source: Nano Converg. 2019 Feb 2;6:4. doi: 10.1186/s40580-019-0174-5 (PMC6358631; doi:10.1186/s40580-019-0174-5)
Supplement: Supplementary file 1 — Additional file 1. Additional figures and tables. [file 40580_2019_174_MOESM1_ESM.docx]

**Additional information**

**Fe, N, S-codoped carbon frameworks derived from nanocrystal superlattices towards enhanced oxygen reduction activity**

Jinxiang Zou^1^, Biwei Wang^1^, Baixu Zhu^2^, Yuchi Yang^2^, Wenqian Han^1^ and Angang Dong^*,1^

^1^iChem, Shanghai Key Laboratory of Molecular Catalysis and Innovative Materials, and Department of Chemistry, Fudan University, Shanghai 200433, China.

^2^State Key Laboratory of Molecular Engineering of Polymers and Department of Macromolecular Science, Fudan University, Shanghai 200433, China.

^*^Correspondence: agdong@fudan.edu.cn


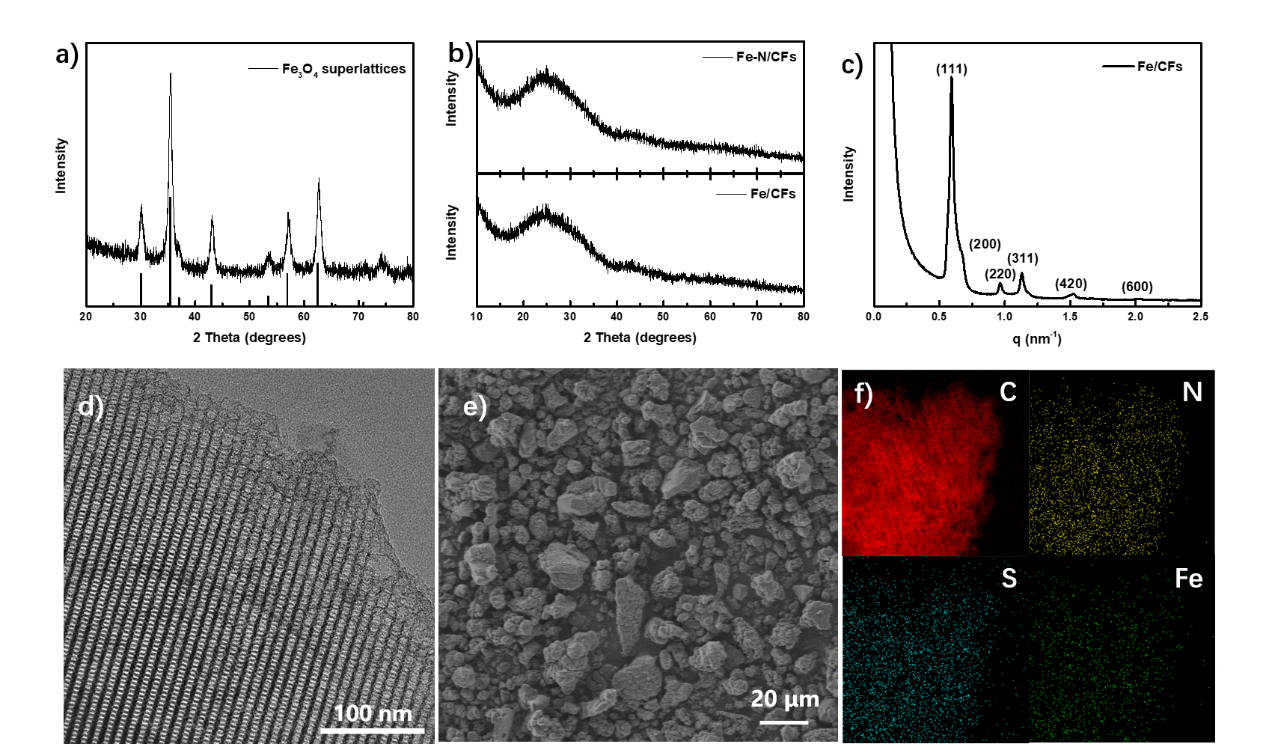


Figure S1. (a) XRD pattern of Fe_3_O_4_ superlattices after heat treatment at 500 °C. (b) XRD patterns of Fe/CFs and Fe-N/CFs. (c) SAXS pattern of Fe/CFs. (d) TEM image of Fe-N-S/CFs. (e) Low-magnification SEM image of Fe-N-S/CFs. (f) EDS elemental mapping of Fe-N-S/CFs.


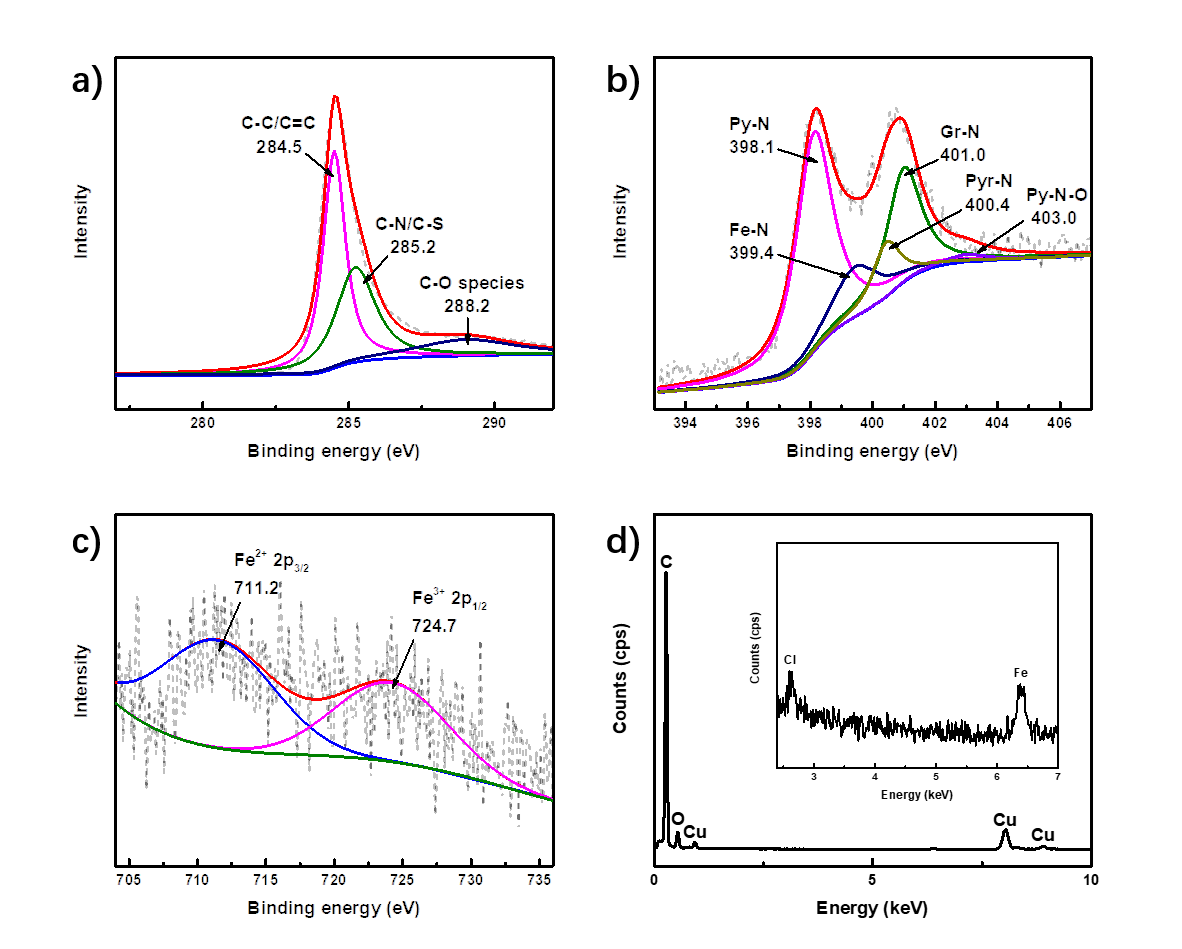


Figure S2. High-resolution (a) C 1s, (b) N 1s and (c) Fe 2p XPS spectra of Fe-N/CFs. (d) EDX spectrum of Fe-N/CFs and the enlarged region between 2.4 and 7.0 keV (insert) which shows a trace amount of Cl existing in the carbon frameworks.


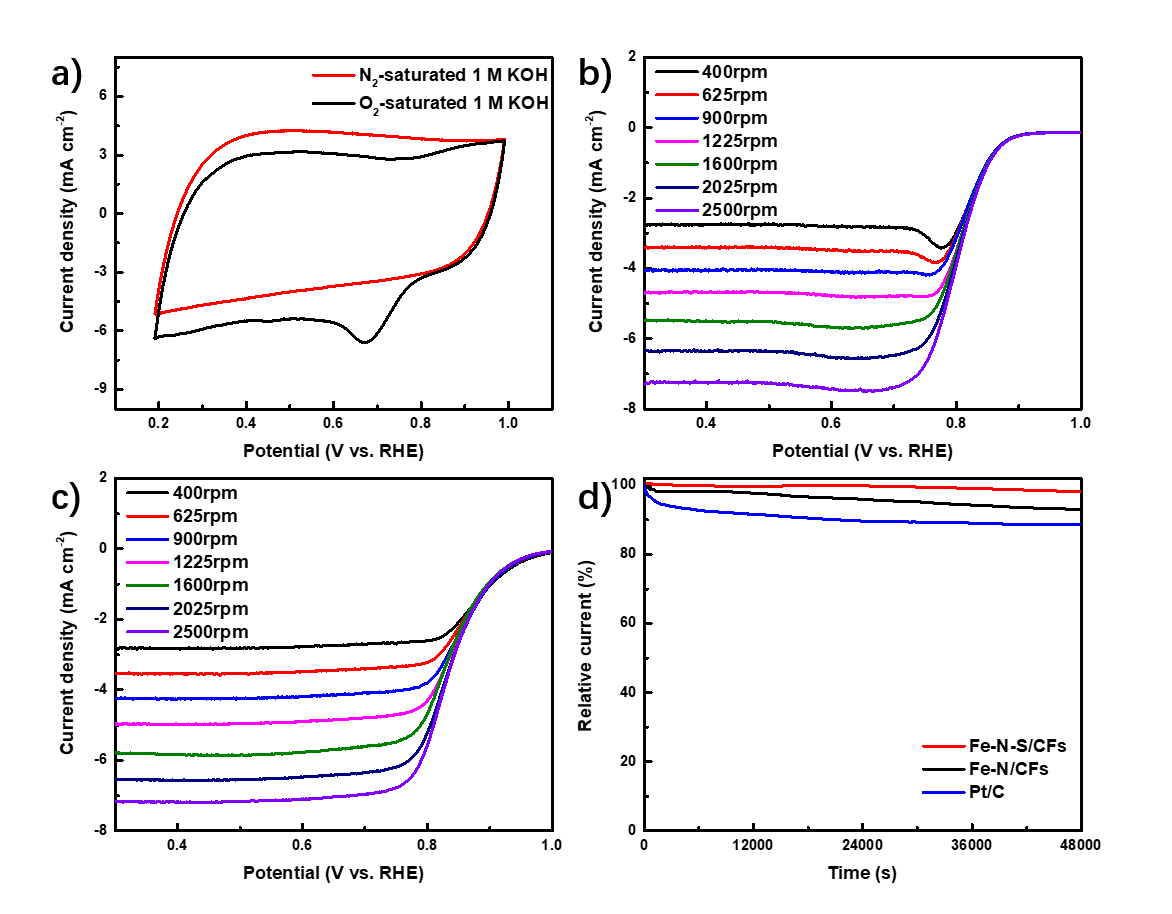


Figure S3. (a) CV curves of Fe-N/CFs tested in O_2_ or N_2_ saturated 0.1 M KOH. (b) LSV curves of Fe-N/CFs with rotating speed ranging from 400 to 2500 rpm. (c) LSV curves of Pt/C with rotating speed ranging from 400 to 2500 rpm. (d) Chronoamperometric measurements of Fe-N-S/CFs, Fe-N/CFs and Pt/C.


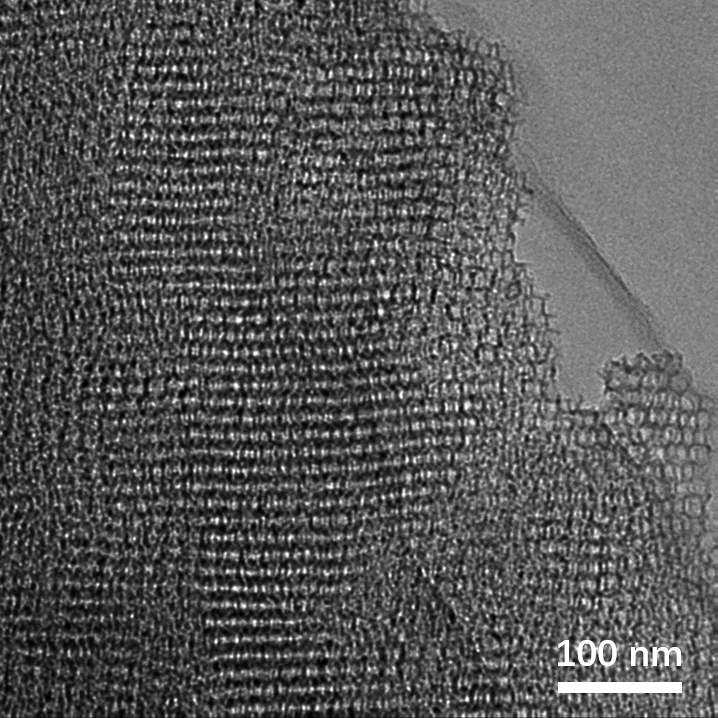


Figure S4. TEM image of Fe-N-S/CFs after long-term durability test.


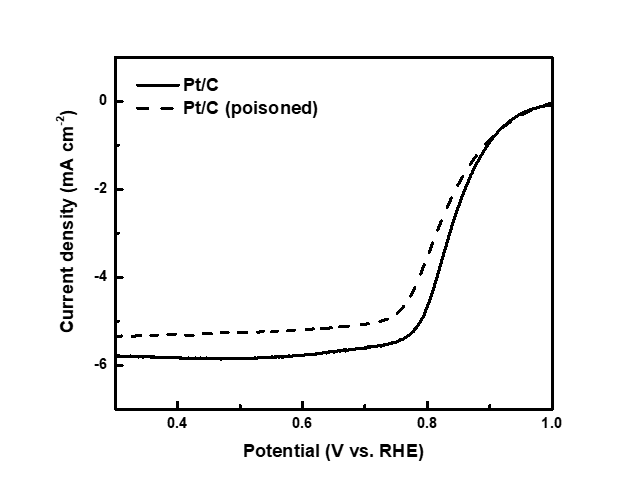


Figure S5. LSV curves of Pt/C measured before and after SCN^-^ poisoning test.


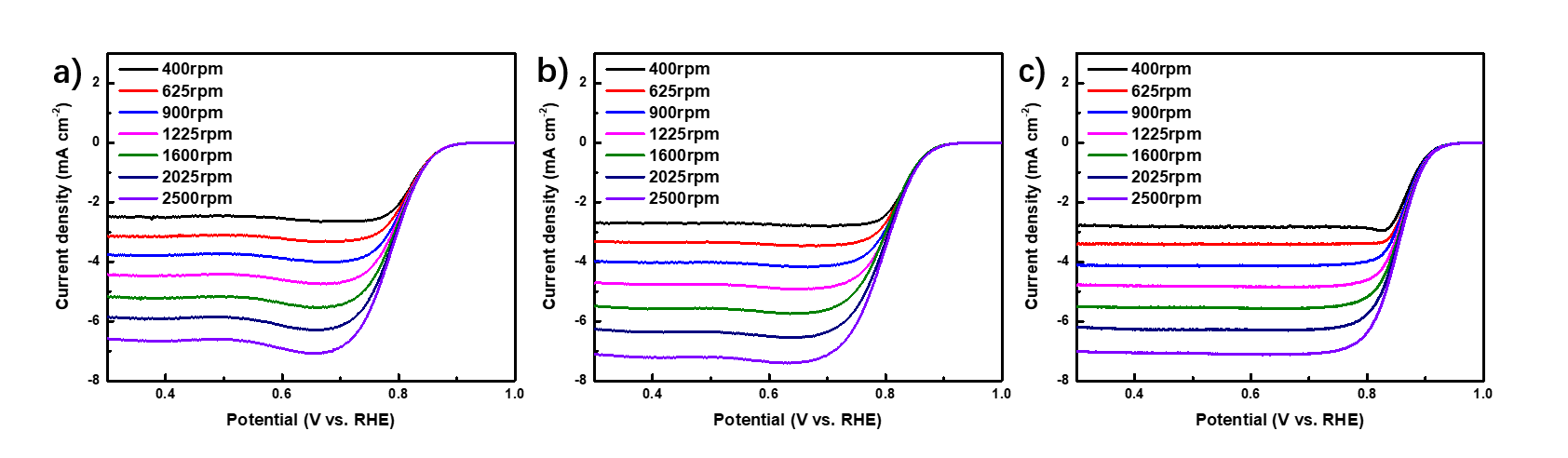


Figure S6. RDE measurements of N and S codoped carbon frameworks doping with mass ratio of (a) 1:0.2, (b) 1:1 and (c) 1:20.

Table S1. Element content of three carbon frameworks measured by EDS.

| Material | Element content / wt% | | | | |
| --- | --- | --- | --- | --- | --- |
|  | C | O | N | S | Fe |
| Fe/CFs | 96.99 | 2.85 | - | - | 0.16 |
| Fe-N/CFs | 93.99 | 2.25 | 3.65 | - | 0.11 |
| Fe-N-S/CFs | 91.36 | 1.36 | 4.18 | 2.97 | 0.14 |

Table S2. Contents (at%) of different types of N doped in Fe-N-S/CFs and Fe-N/CFs.

|  | Py-N | Fe-N | Pyr-N | Gr-N | Py-N-O |
| --- | --- | --- | --- | --- | --- |
| Fe-N-S/CFs | 0.63 | 1.07 | 0.62 | 1.08 | 0.30 |
| Fe-N/CFs | 1.58 | 0.46 | 0.30 | 0.77 | 0.05 |

Table S3. N and S contents doped in carbon frameworks at different mass ratios.

| Mass ratio  (carbon frameworks:thiourea) | N  (wt%) | S  (wt%) |
| --- | --- | --- |
| 1:0.2 | 2.96 | 1.05 |
| 1:1 | 3.77 | 1.65 |
| 1:10 | 4.18 | 2.97 |
| 1:20 | 4.96 | 4.76 |
